# Ulcerative colitis successfully treated with vedolizumab in the presence of comorbid opportunistic infections: a case report

**DOI:** 10.1186/s13256-023-03940-y

**Published:** 2023-05-31

**Authors:** Yusuke Oki, Sho Nagano, Yoichi Ishikawa, Takayoshi Yamada, Toshiki Ichimori, Kazushige Uchida

**Affiliations:** 1 Department of Gastroenterology and Hepatology, Kochi Medical School, Kochi University, Kohasu, Oko-Cho, Nankoku, Kochi 783-8505 Japan; 2 Department of Gastroenterology, Kochi Health Sciences Center, Kochi, Japan; 3 Department of Internal Medicine, Susakikuroshio Hospital, Susaki, Japan

**Keywords:** Ulcerative colitis, Vedolizumab, Opportunistic infection, Cryptococcal pneumonia, Cytomegalovirus

## Abstract

**Background:**

Opportunistic infections associated with immunosuppressive treatments for inflammatory bowel disease pose an important safety concern. Here we report the case of a patient with active ulcerative colitis and cryptococcal pneumonia who was treated with vedolizumab combined with fluconazole.

**Case presentation:**

A 56-year-old Japanese man with ulcerative colitis and a history of Sweet’s syndrome who was taking prednisolone and azathioprine presented with a moderate exacerbation of ulcerative colitis, abdominal pain, diarrhea, and bloody stools along with cytomegalovirus infection. Increasing the prednisolone dose without using antiviral drugs improved cytomegalovirus infection; however, ulcerative colitis did not improve, and cryptococcal pneumonia occurred. Thus, treatment with fluconazole followed by vedolizumab was initiated for ulcerative colitis. The patient gradually recovered and achieved clinical remission without the exacerbation of pneumonia.

**Conclusions:**

We reported the first case of a patient with ulcerative colitis who was treated with vedolizumab and concomitant fluconazole for active cryptococcal pneumonia. Vedolizumab constitutes a high-potential treatment regimen owing to its safety in inflammatory bowel disease associated with opportunistic infections.

## Background

The role of immunosuppressive treatments in inflammatory bowel disease (IBD) is currently becoming increasingly important, and opportunistic infections associated with these treatments are an important safety concern [1]. Vedolizumab (VDZ) is a biological agent targeting α4β7 integrin, which exerts positive effects in adult patients with moderate-to-severe active ulcerative colitis (UC) or Crohn’s disease (CD) [2–4]. VDZ is expected to be highly safe owing to its gut selectivity. However, the safety of VDZ administration to patients with IBD with opportunistic infections remains unclear. Here we report the case of a patient with active UC and cryptococcal pneumonia who was treated with VDZ combined with fluconazole, showing a good treatment course.

## Case presentation

**Fig. 1:**
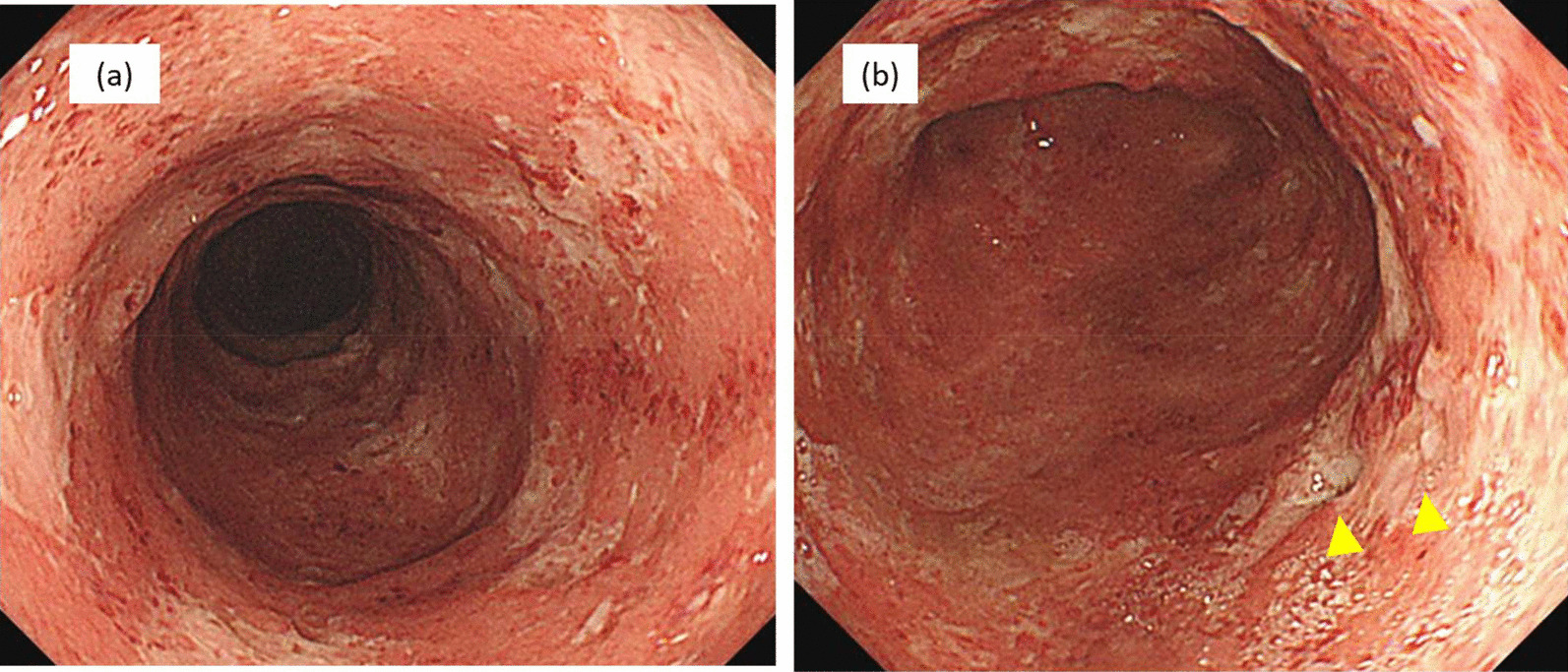
Colonoscopy findings on admission. **a** Diffuse mucosal inflammation extending from the lower rectum to transverse colon. **b** Several ulcerations (arrowheads) in the descending and sigmoid colon

A 56-year-old Japanese man with UC (pancolitis type) was admitted to our hospital for exacerbated UC associated with abdominal pain, diarrhea, and bloody stools that had lasted for a month. His past medical history was remarkable for hypertension, dyslipidemia, and Sweet’s syndrome. He had no specific family history or obvious history of infection, surgery, and psychosocial background. He smoked 10 cigarettes daily until his 30s and was an occasional drinker. He was diagnosed with UC approximately 10 months before hospital admission, and treatment with mesalamine (4800 mg once daily) and prednisolone (PSL, 30 mg/day) was started orally for UC at that time. Following the recovery of the patient, the PSL dose was tapered off (tapered by 5 mg/day every 2 weeks up to 10 mg/day and then by 2.5 mg/day), whereas oral mesalamine was continued (4800 mg once daily). At 3 months following UC diagnosis, after reducing the PSL dose from 10 mg/day to 7.5 mg/day, the patient developed pyrexia and skin rashes and was diagnosed with Sweet’s syndrome. The PSL dose was subsequently increased to 30 mg/day, after which the patient recovered, and the PSL dose was further tapered off gradually. Oral mesalamine was discontinued owing to mesalamine intolerance; thus, azathioprine (50 mg/day) was introduced to maintain the clinical remission of UC. Upon admission, the patient was taking PSL (3 mg/day) and azathioprine (75 mg/day). On examination, his body temperature, heart rate, and blood pressure level were 36.8 °C, 80 beats per minute, and 103/73 mmHg, respectively. Further, he presented with tenderness on the left side of the abdomen. His blood test results revealed slightly elevated C-reactive protein (CRP) levels and erythrocyte sedimentation rates (Table 1). Colonoscopy findings revealed diffuse mucosal inflammation extending from the lower rectum to transverse colon as well as several ulcerations in the descending and sigmoid colon (Fig. 1). Stool culture results revealed no abnormal findings.

**Table 1:** Laboratory Findings on Admission

WBC	6400/µl		Tp	7 g/dL	CRP	4.78 mg/dL	
Neut	56.6%		Glu	123 mg/dL	Na	133 mEq/L	
Lym	22.7%		ALP	164U/L	K	4.4 mEq/L	
Mono	10.6%		γ-GT	40U/L	Cl	101 mEq/L	
Eo	9.5%		T-bil	0.7 mg/dL			
Bas	0.6%		Alb	3.8 g/dL	ESR	41 mm/h	
			ALT	24U/L			
RBC	444 × 10^4^/µL		AST	25 U/L	CMV-Ag	16 cells/50,000 WBC	
Hb	13.8 g/dL		LDH	189U/L	(HRP-C7 method)		
Ht	40.5%		Cr	1.12 mg/dL			
Plt	33 × 10^4^/µL		BUN	18.2 mg/dL			
			Amy	69 U/L			

*γ-GT* gamma-glutamyl transpeptidase, *Alb* albumin, *ALP* alkaline phosphatase, *ALT* alanine aminotransferase, *Amy* amylase, *AST* aspartate aminotransferase, *Bas* basophil, *BUN* blood urea nitrogen, *Cl* chlorine, *CMV-Ag* cytomegalovirus antigenemia, *Cr* creatinine, *CRP* C-reactive protein, *Eo* eosinophil, *ESR* erythrocyte sedimentation rate, *Glu* glucose, *Hb* hemoglobin, *Ht* hematocrit, *K* potassium, *LDH* lactate dehydrogenase, *Lym* lymphocyte, *Mono* monocyte, *Na* sodium, *Neut* neutrophil, *Plt* platelet, *RBC* red blood cell, *T-bil* total bilirubin, *TP* total protein, *WBC* white blood cell

The patient was diagnosed with moderately exacerbated UC, and PSL was initiated with an increasing dose up to 30 mg/day. Pathological biopsy samples obtained from colonic ulcers were found to be positive for cytomegalovirus (CMV) immunohistochemistry (IHC) and blood cytomegalovirus-antigenemia (CMV-Ag) using the HRP-C7 method; further, 16/50,000 white blood cells and a few inclusion bodies were detected, indicating CMV infection (reactivation). However, the patient exhibited mild improvement after the administration of increasing doses of PSL. Furthermore, a previous study reported that PSL treatment alone can resolve UC-associated CMV reactivation during immunosuppressive therapy without the need for antiviral drugs [5]; therefore, we continued PSL treatment in combination with granulocyte and monocyte adsorption apheresis (GMA) without antiviral drugs such as ganciclovir.

**Fig. 2:**
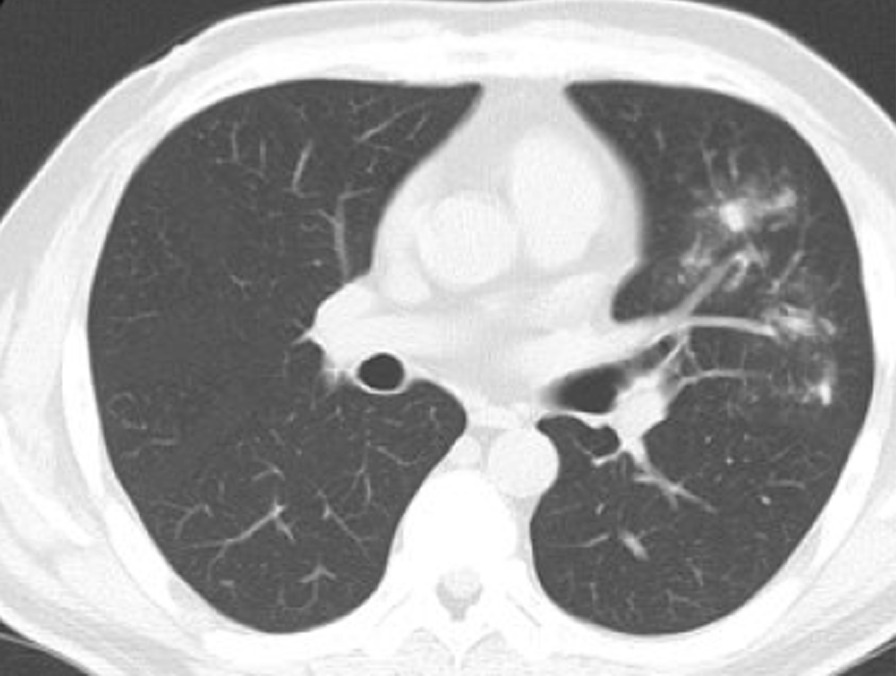
Chest computed tomography after PSL administration (10 mg/day). At 41 days after the administration of increased doses of PSL (when the PSL dose reached 10 mg/day), chest computed tomography revealed infiltrative and granular shadows in the left lung. *PSL* prednisolone

**Fig. 3:**
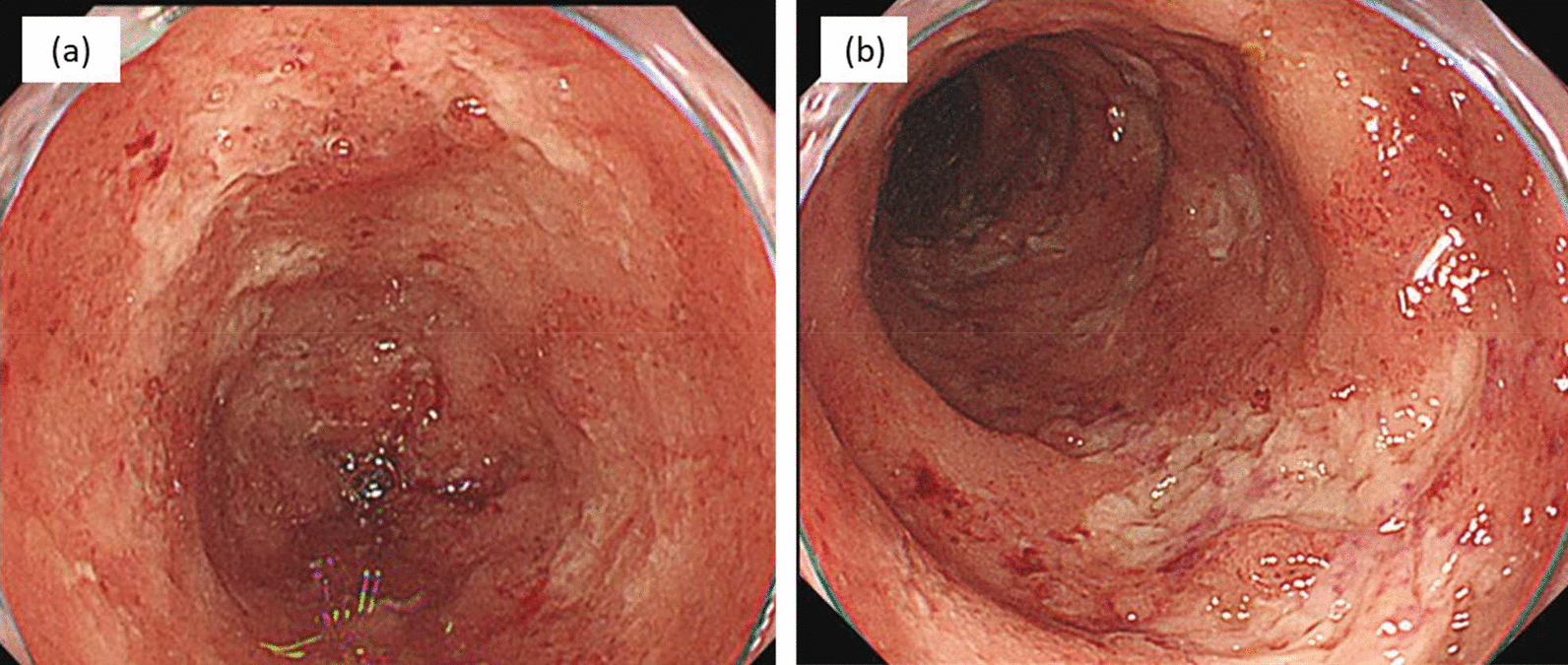
Colonoscopy findings 19 days after PSL discontinuation. Colonoscopy 19 days after PSL discontinuation showed worsening findings with ulcers in the descending and sigmoid colon. CMV immunohistochemistry of the colonic mucosa became negative. **a** Transverse colon and **b** descending colon. *CMV* cytomegalovirus, *PSL* prednisolone

Subsequently, his condition improved, and he was discharged from the hospital. His PSL dose was further tapered off. However, when the PSL dose reached 15 mg/day, his condition gradually worsened again with symptoms such as diarrhea, abdominal pain, and gradual elevation of serum CRP levels. At 41 days after the administration of increased doses of PSL (when the PSL dose reached 10 mg/day), chest computed tomography (CT) revealed infiltrative and granular shadows in the left lung (Fig. 2). An additional laboratory test revealed that the patient was positive for serum cryptococcal antigen. Consequently, he was diagnosed with cryptococcal pneumonia and started receiving fluconazole. Colonoscopy performed on the same day as chest CT revealed that his colitis had not improved and CMV IHC of the colonic mucosa remained positive, although blood CMV-Ag was already negative. The PSL dose was then tapered off the next week; however, fever and bloody stools appeared after PSL discontinuation. Colonoscopy at 19 days after PSL discontinuation indicated worsening of the condition, with ulcers in the descending and sigmoid colon (Fig. 3), although CMV IHC of the colonic mucosa became negative. No pneumonia exacerbation was observed on chest CT. On the basis of these findings, we considered that these colonoscopy findings were due to exacerbated UC, not CMV. As VDZ is a gut-selective antibody against α4β7 integrin with relatively fewer side effects [2], we used it as an additional treatment for UC. This is because the treatment for cryptococcal pneumonia was started at that time and CMV infection had been cured.

**Fig. 4:**
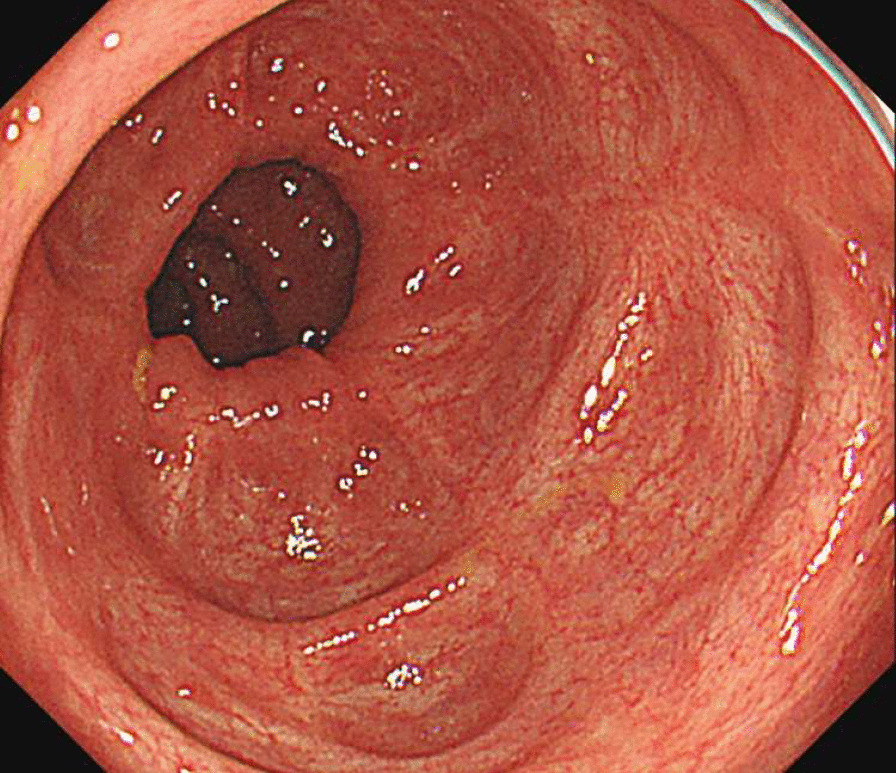
Colonoscopy findings in the transverse colon after 17 months of VDZ administration. A colonoscopy after 17 months of VDZ administration revealed mucosal healing. *VDZ* vedolizumab

**Fig. 5:**
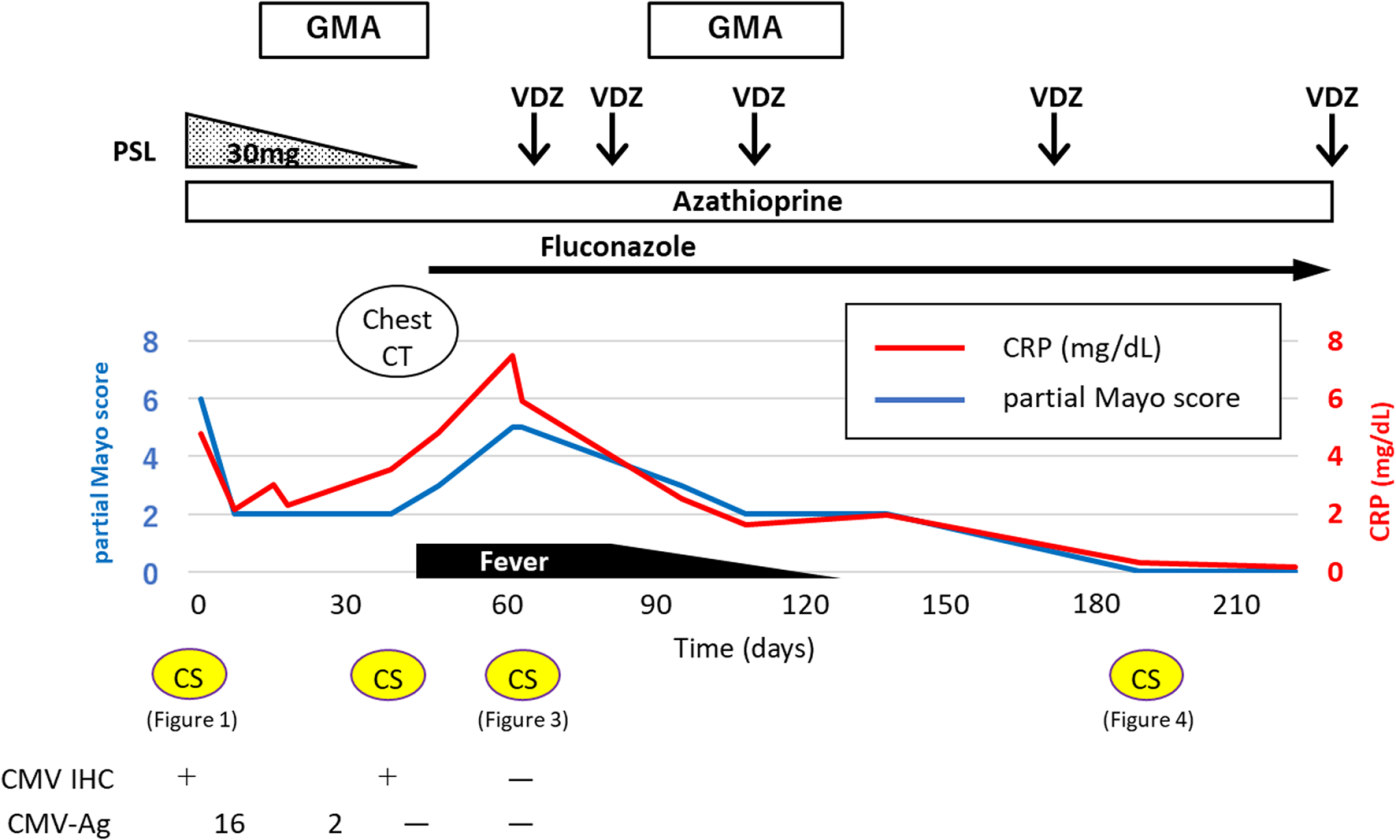
The clinical course from the time of the second steroid dose increase. “0 day” indicates the day the PSL dose was increased to 30 mg/day. The clinical activity in UC is shown using the partial Mayo score [6], which consisted of three Mayo subscores, excluding an endoscopic subscore. A moderate UC exacerbation with CMV infection developed, which was treated with PSL and GMA. Although CMV infection was cured, colitis did not improve, and cryptococcal pneumonia occurred. After fluconazole administration, VDZ was initiated for UC. *CMV* cytomegalovirus, *CMV-Ag* blood CMV antigenemia, *CRP* C-reactive protein, *CT* computed tomography, *CS* colonoscopy, *GMA* granulocyte and monocyte adsorption apheresis, *IHC* immunohistochemistry; *PSL* prednisolone, *UC* ulcerative colitis, *VDZ* vedolizumab

Subsequently, VDZ (300 mg/body) combined with fluconazole was initiated, and GMA was restarted after 2 weeks of VDZ administration. Consequently, his symptoms, such as frequent episodes of fever, diarrhea, abdominal pain, and elevated serum CRP levels, were alleviated. Colonoscopy performed 4 months after VDZ administration revealed a clear improvement in colonic and rectal inflammation, with residual slight ulcers and erosions in the colon. No pneumonia exacerbation was observed on chest X-ray and CT. Fluconazole was continued for 6 months, and the treatment course was completed after confirming no exacerbation of pneumonia. Colonoscopy performed 17 months after VDZ administration revealed mucosal healing (Fig. 4). Currently, the patient is receiving VDZ every 8 weeks and has maintained clinical remission (Fig. 5); he could continue his treatment without facing any difficulty in his daily life.

## Discussion and conclusions

VDZ is a gut-selective antibody targeting α4β7 integrin, which exerts positive effects in adult patients with moderate-to-severe active UC or CD [2–4]. Lymphocytes expressing α4β7 integrin specifically bind to mucosal address in cell adhesion molecule-1 expressed on the vascular endothelial cell surface in the gastrointestinal tract and migrate to the gastrointestinal tract [2, 7]. VDZ inhibits this binding and selectively blocks lymphocyte migration to the gut, consequently indicating few risks of opportunistic infections [8].

The incidence rate of opportunistic infections associated with VDZ was 0.7/100 patient-years (PY) in GEMINI 1 and 2 and 1.0/100 PY in GEMINI LTS [9]. The frequent opportunistic infections reported in these studies were *Clostridium difficile* (*C*. *difficile*) and CMV infections. In GEMINI 1 and 2, the incidence rates of *C*. *difficile* colitis, CMV colitis, and CMV infection were 0.5, 0.1, and 0.1/100 PY, respectively. Similarly, in GEMINI LTS, the incidence rates of *C*. *difficile* and CMV colitis were 0.5 and 0.1/100 PY, respectively. Most of these opportunistic infections were not serious, and it was adequately safe to continue VDZ. In the postmarketing setting of approximately 114,071 PY of VDZ therapy, opportunistic infections were reported in 210 patients (217 events). The most common events reported in these patients were also *C*. *difficile* (83 non-serious and 44 serious events) and CMV (6 non-serious and 14 serious events) infections [9]. Furthermore, another study reported that VDZ did not increase the risk of serious and opportunistic infections compared with placebo [10]. Thus, VDZ can be considered safe for use.

We searched PubMed for patients with IBD treated with VDZ in the presence of opportunistic infections and identified eight patients (five with UC and three with CD) [11–17], including the present case (Table 2). Opportunistic infections in these patients included pneumonia with *Staphylococcus haemolyticus* (one patient), pneumocystis pneumonia (one patient), herpes simplex virus infection (one patient), CMV infection (seven patients), and cryptococcal pneumonia (two patients). One patient [13] had human immunodeficiency virus infection along with CMV pneumonia, pneumocystis pneumonia, and herpes simplex viral infection. CMV infection was observed in seven patients (including our case), and seven of eight patients were under immunosuppressive treatment. On the basis of the patient records, five patients started VDZ therapy following treatment for opportunistic infections. In one patient [12], the details regarding opportunistic infections were unavailable. One of the remaining two patients had active UC and CMV infection and recovered after VDZ treatment without the need for antiviral therapy, although CMV infection was detected after VDZ administration [14]. The other case was of a patient with UC and primary CMV infection who was receiving VDZ and azathioprine before primary CMV infection. After primary CMV infection, he stopped receiving azathioprine and recovered with the use of an antiviral drug (ganciclovir) without VDZ discontinuation [16]. None of the patients had exacerbated opportunistic infections, and six of eight patients achieved remission. However, one patient [15] required surgical resection 7 months after VDZ initiation due to UC exacerbation, although the patient was in the clinical remission phase while receiving VDZ. Our patient with UC and cryptococcal pneumonia showed significant responses to VDZ combined with fluconazole, and no pneumonia exacerbation was noted after initiating VDZ. Furthermore, on the basis of our literature search via PubMed, there was only one case [11] other than the present case where VDZ was used for treating diseases with coexisting cryptococcal infection (Table 2). In that case [11], a patient with CD and cryptococcal pneumonia was administered adalimumab and was successfully treated with antifungal agents. When the patient was receiving adalimumab treatment, a rectovaginal fistula appeared, and he underwent transverse colostomy. The medication was then changed to VDZ, and the patient achieved clinical remission after 2 months. Conversely, in our case, VDZ was started concurrently with the treatment for cryptococcal pneumonia, and neither UC nor pneumonia worsened. To the best of our knowledge, this is the first case where VDZ was initiated in a patient who was receiving treatment for active cryptococcal pneumonia.

**Table 2:** Summary of eight cases of IBD using VDZ in the presence of opportunistic infections

Reference	Age	Gender	Years	IBD	Opportunistic infections	Other comorbidities	Activity of IBD before VDZ	Treatment of IBD before VDZ	Treatment of opportunistic infections	Status of opportunistic infections at VDZ initiation	Follow-up after VDZ
Our case	56	M	2022	UC	Cryptococcal pneumonia CMV infection	History of Sweet's syndrome	Moderate exacerbation	PSL, AZA, and GMA	Fluconazole	under treatment (Cryptococcal pneumonia)	Remission
11	57	F	2021	CD	Cryptococcal pneumonia CMV infection	SLE	Existence of rectovaginal fistula	PSL, ADA, hydroxychloroquine, and transverse colostomy	Valganciclovir, amphotericin B and flucytosine	Cured by treatments	Remission, but the fistula persisted
12	74	M	2017	UC	Pneumonia with *Staphylococcus haemolyticus*	Hemophilia A, coronary artery disease, type 2 DM, and PSC	Remission	Mesalamine, IFX, and history of using 6-mercaptopurine	NA	NA	Remission
13	30	M	2019	CD	CMV pneumonia, pneumocystis pneumonia, and herpes simplex virus infection	HIV infection	Increased bowel movement frequency and elevated CRP	History of using ADA	NA	Cured by treatments	Remission
14	18	F	2019	UC	CMV infection	TPMT deficiency	NA	PSL	None	Positive for CMV immunostain on colon biopsy	Remission
15	21	M	2020	UC	CMV infection	NA	Flare	Steroid, IFX, and history of using GLM	GCV	Cured by treatments	Remission, but then flared and required proctocolectomy
16	33	M	2020	UC	CMV infection	PSC	Flare	IFX, ADA, and GLM	GCV and discontinuation of AZA	Occurred during VDZ administration	Response+
17	47	M	2021	CD	CMV infection	Liver cirrhosis (due to HCV and alcoholism)	Multiple rectal fistulas and presacral abscess	Antibacterial drug and transverse colostomy	GCV and valganciclovir	Under treatment	Remission

*ADA* adalimumab, *ART* antiretroviral therapy, *AZA* azathioprine, *CD* Crohn’s disease, *CMV* cytomegalovirus, *CRP* C-reactive protein, *DM* diabetes mellitus, *GCV* ganciclovir, *GMA* granulocyte and monocyte adsorption apheresis, *GLM* golimumab, *HCV* hepatitis C virus, *HIV* human immunodeficiency virus, *IBD* inflammatory bowel disease, *IFX* infliximab, *mPSL* methylprednisolone, *NA* not available, *PSC* primary sclerosing cholangitis, *PSL* prednisolone, *SLE* systemic lupus erythematosus, *TPMT* thiopurine S-methyltransferase, *UC* ulcerative colitis, *VDZ* vedolizumab

It is well known that CMV reactivation is related to UC refractoriness [18]; however, in our case, VDZ was effective and achieved mucosal healing despite the severe endoscopic activity of the colon and accompanying CMV infection. This may be because CMV infection improved during VDZ initiation. However, we consider that VDZ initiation as first biologic was responsible for this effect because a previous study reported that the remission rate of VDZ in bio-naïve patients was higher than that in patients with prior exposure to antitumor necrosis factor-α antagonist [19]. In addition, distinguishing CMV colitis from UC flare ups with CMV reactivation is challenging in some cases. Herein, colorectal pathology and positive findings of blood CMV-Ag upon admission indicated CMV colitis. However, CMV reactivation could occur during UC flare ups, and such conditions often improve even without antiviral treatment [5]. The symptoms of our patient improved following the administration of increased doses of PSL. Furthermore, the CMV-Ag and pathological findings of CMV reactivation on colonic mucosal biopsy improved when the symptoms worsened during PSL dose tapering. The patient continued to receive UC treatment (including PSL and VDZ) without antiviral therapy; subsequently, his symptoms and endoscopic findings improved, and CMV infection was cured. Thus, we suggest that UC exacerbation rather than CMV was associated with the worsening of symptoms in our patient.

Herein, we reported the first case of a patient with UC treated with VDZ and concomitant fluconazole for active cryptococcal pneumonia. Thus, VDZ is a highly feasible and safe treatment option for IBD associated with opportunistic infections. Because our case involved a single patient, further analysis with more cases is expected.

## Data Availability

All data generated or analyzed during this study are included in this published article.

## Abbreviations

CD: Crohn’s disease
CMV: Cytomegalovirus
CMV-Ag: Cytomegalovirus-antigenemia
CT: Computed tomography
CRP: C-reactive protein
GMA: Granulocyte and monocyte adsorption apheresis
HIV: Human immunodeficiency virus
IBD: Inflammatory bowel disease
IHC: Immunohistochemistry
PSL: Prednisolone
PY: Patient-years
UC: Ulcerative colitis
VDZ: Vedolizumab
